# Evaluation of Factors Affecting Neuropathy in Patients With Type 2 Diabetes Using Artificial Neural Networks

**DOI:** 10.7759/cureus.61860

**Published:** 2024-06-06

**Authors:** Jamileh Abolghasemi, Shahnaz Rimaz, Sadegh Kargarian-Marvasti

**Affiliations:** 1 Department of Biostatistics, School of Public Health, Iran University of Medical Sciences, Tehran, IRN; 2 Radiation Biology Research Center, Iran University of Medical Sciences, Tehran, IRN; 3 Centers for Disease Control and Prevention, Health Center of Fereydunshahr, Isfahan University of Medical Sciences, Isfahan, IRN

**Keywords:** cohort study, auc, artificial neural networks, neuropathy, diabetes type 2

## Abstract

Introduction: Neuropathy is a common and debilitating complication in type 2 diabetes, affecting quality of life and increasing healthcare costs. Identifying risk factors is essential for early intervention and management. This study aims to evaluate the factors influencing the occurrence of neuropathy in patients with type 2 diabetes using artificial neural networks.

Methods: In this cohort study, data from 371 patients with type 2 diabetes from Fereydunshahr, Iran, were analyzed over a 12-year follow-up period. Participants were selected based on diabetes screenings conducted in 2008 and 2009. Artificial neural networks with varying architectures were trained and validated, and their performance was compared to logistic regression models using receiver operating characteristic (ROC) curve analysis.

Results: The prevalence of neuropathy in this cohort study was 31.2%. The best-fitted artificial neural network and logistic regression model had area under the curve (AUC) values of 0.903 and 0.803, respectively. Significant risk factors identified included gender, race, family history of diabetes, type of diabetes treatment, cholesterol levels, triglyceride levels, high-density lipoprotein (HDL) levels, and duration of diabetes. Notably, women, patients with a family history of diabetes, and those using injectable or combined injectable and oral medications were at higher risk of developing neuropathy.

Conclusion: These findings highlight the importance of vigilant monitoring and proactive management of neuropathy risk factors, especially in women, patients with a family history of diabetes, and those using injectable or combined diabetic medications.

## Introduction

Diabetes mellitus, particularly type 2 diabetes mellitus (T2DM), represents a significant and growing public health challenge globally. As of 2019, approximately 463 million adults were living with diabetes, a number expected to surge to 700 million by 2045 [1]. This chronic condition not only diminishes the quality of life for individuals but also imposes a considerable economic burden on healthcare systems worldwide. Direct medical costs, including hospitalizations, medications, and outpatient care, along with indirect costs such as lost productivity and disability, make diabetes one of the most expensive chronic diseases. In 2017, the global expenditure on diabetes was estimated at $727 billion, highlighting the extensive financial impact of the disease [2]. Addressing diabetes and its complications is therefore crucial for improving public health outcomes and reducing economic strain.

Diabetes mellitus leads to a range of severe complications, significantly impacting the health and quality of life of affected individuals. Among these complications, diabetic neuropathy is particularly prevalent and debilitating, affecting approximately 50% of patients with diabetes during their lifetime [3]. Diabetic neuropathy encompasses a variety of nerve disorders caused by diabetes, with peripheral neuropathy being the most common form. This condition primarily results from prolonged hyperglycemia, which leads to nerve damage, manifesting as pain, numbness, and weakness, especially in the hands and feet. Beyond peripheral neuropathy, diabetes can also cause autonomic neuropathy, which affects the autonomic nervous system and can lead to symptoms such as gastrointestinal issues, cardiovascular problems, and sexual dysfunction. These complications not only cause significant morbidity but also increase the risk of mortality among diabetic patients [4]. The substantial impact of diabetic neuropathy on patient well-being and healthcare resources underscores the importance of understanding its risk factors to develop effective prevention and management strategies.

Given the increasing prevalence and substantial burden of T2DM and its complications, understanding the risk factors for diabetic neuropathy is of paramount importance. Diabetic neuropathy not only significantly diminishes the quality of life for affected individuals but also contributes to higher healthcare costs and increased morbidity and mortality [3,4]. Identifying the factors that predispose patients to this condition can facilitate early intervention, improve patient outcomes, and optimize the allocation of healthcare resources.

Neural networks, a subset of machine learning and artificial intelligence, have garnered significant attention for their ability to model complex, non-linear relationships within data. Unlike traditional statistical methods, which often rely on predefined assumptions about data distribution and relationships, neural networks can learn directly from the data itself, adapting to patterns and nuances that might be missed by conventional techniques [5]. This adaptability makes them particularly powerful for handling large and diverse datasets. Neural networks excel in feature extraction and pattern recognition, which are crucial for identifying subtle interactions between risk factors and outcomes [6]. Their layered architecture allows for the progressive transformation of raw input data into higher-level abstractions, enabling more accurate predictions and insights [7]. Furthermore, advancements in computational power and algorithm optimization have made neural networks more accessible and efficient, reducing computational costs and processing times [8]. These advantages position neural networks as a superior tool for predictive modeling in complex domains such as healthcare, where understanding multifaceted interactions between variables is essential for improving patient outcomes and developing targeted interventions [9].

This study aims to evaluate the risk factors for neuropathy in patients with T2DM using advanced analytical methods, specifically neural networks. Neural networks, as sophisticated computational models, have the potential to uncover complex patterns and interactions within large datasets that traditional statistical methods might overlook.

## Materials and methods

In this cohort study, data from 371 patients with T2DM were analyzed. Adults who tested negative for diabetes during the 2008 diabetes screening in Fereydunshahr County (the westernmost county of Isfahan Province in Iran) and were diagnosed with diabetes during the 2009 screening were included in the study after obtaining written informed consent. Their vital status and diabetes-related complications were followed up for a period of 12 years.

The study was approved by the Ethics Committee of Iran University of Medical Sciences (approval number: IR.IUMS.REC.1398.322). All participants provided informed written consent before participating in the study. They were reminded that participation was voluntary and confidential and that the results would remain anonymous. Appropriate ethical principles and methods were strictly adhered to during the collection of data. We ensured that all procedures involving human subjects complied with the ethical standards outlined. Anonymity/confidentiality was maintained throughout the study.

Inclusion and exclusion criteria

Participants included individuals aged 30 and older, residing in Fereydunshahr, Isfahan, and diagnosed with T2DM. They were selected based on diabetes screenings conducted in 2008 and 2009, where their test results were negative in 2008 and positive in 2009. At the beginning of the study, they did not have neuropathy. The exit criterion was patient dissatisfaction with continuing participation in the study.

Measures

The response variable was the incidence of neuropathy. Information on variables such as demographic factors (gender, age, occupation, education, place of residence, race), family history of diabetes in first-degree relatives, diabetes treatment method, smoking status, and laboratory variables (including fasting blood sugar (FBS), cholesterol, triglycerides, high-density lipoprotein (HDL), low-density lipoprotein (LDL), creatinine, glycated hemoglobin (HbA1c)), body mass index (BMI), diastolic blood pressure, and systolic blood pressure), as well as clinical and questionnaire data related to neuropathy and neuropathy diagnosis using nerve conduction studies, was recorded by reviewing the patients' records and conducting interviews when necessary.

Statistical analysis

After data collection and cleaning, the normality of quantitative variables was assessed using the Kolmogorov-Smirnov test. To compare demographic and clinical variables between the neuropathy and non-neuropathy groups, Chi-square tests, Fisher's exact test, and independent t-tests were used. Artificial neural networks (ANNs) and multiple logistic regression were employed to examine factors influencing neuropathy. Simple logistic regression was conducted at a significance level of 0.2, while multiple logistic regression and other tests were performed at a significance level of 0.05. Data analysis was carried out using SPSS for Windows, Version 13.0 (SPSS Inc., Chicago, United States) and R software version 4.3.2 (R Foundation for Statistical Computing, Vienna, Austria).

To fit the ANN, initially, the data was randomly divided into two groups of training and testing with a ratio of 70 to 30. Then, the neural network was fitted with one hidden layer, followed by fitting it with two hidden layers. The number of nodes in the second layer was varied between 4 and 12 to select the optimal network. Hyperbolic tangent and sigmoid activation functions were used for the input layer, and the same functions were used for the output layer. To select the optimal ANN, the performance characteristic curve area, percentage of correct predictions, and mean squared error were considered. For conducting multiple logistic regression, initially, simple logistic regression was performed for all variables at a significance level of 0.2. Significant variables were then considered as candidates for entry into the multiple logistic regression model, which was fitted using the forward method at a significance level of 0.05.

## Results

The results of comparing gender distribution, education level, ethnicity, occupational activity, cardiovascular diseases, smoking, family history of diabetes, and treatment type between patients with and without neuropathy are presented in Table 1. The distribution of gender, race, family history of diabetes, and type of treatment differed between the neuropathy and non-neuropathy groups. Patients with neuropathy were more likely to be female, of Persian ethnicity, have a family history of neuropathy, and receive injectable or combined injectable and oral medications.

**Table 1:** Demographic variables of diabetic patients by neuropathy status τ: Fisher exact test, *X^2^: *Statistic of Chi-square test or Fisher exact test

Variables	Categories	Neuropathy+	Neuropathy-	X^2^	P-value
Gender	Male	26 (22.4%)	88 (34.5%)	5.480	0.019
	Female	90 (77.6%)	167 (65.5%)		
Education	Illiterate	87 (75.0)	170 (70.2)	2.234 ^τ^	0.536
	Elementary School	26 (22.4)	56 (23.1)		
	High School	2 (1.7)	10 (4.0)		
	Diploma	1 (0.9)	6 (2.5)		
Race	Georgian	36 (31.0%)	101 (39.8%)	9.263	0.026
	Bakhtiari	32 (27.6%)	85 (33.5%)		
	Persian	18 (15.5%)	19 (7.5%)		
	Turk	30 (25.9%)	49 (19.3%)		
Job-related activity	Low	15 (12.9%)	42 (16.5%)	2.010	0.366
	Moderate	85 (73.3%)	168 (65.9%)		
	High	16 (13.8%)	45 (17.6%)		
Occupation status	Employed	28 (24.3)	75 (30.0)	4.350^ τ^	0.219
	Housewife	82 (71.3)	160 (64.0)		
	Retired	2 (1.7)	12 (4.8)		
	Unemployed	3 (2.6)	3 (1.2)		
Heart Disease	Yes	22 (19.1)	55 (22.5)	2.054	0.382
	No	93 (80.9)	187 (77.5)		
Smoking	Yes	105 (90.5)	230 (92.7)	0.533	0.465
	No	11 (9.5)	18 (7.3)		
Family History	Yes	74 (64.3%)	104 (44.4%)	12.223	<0.001
	No	41 (35.7%)	130 (55.6%)		
Treatment type	Oral	85 (73.3%)	229 (89.8%)	20.813	<0.001
	Insulin	13 (11.2%)	15 (5.9%)		
	Both	18 (15.5%)	11 (4.3%)		
Residential zone	Rural	48 (41.4)	101 (41.2)	0.001	0.978
	Urban	68 (58.6)	144 (58.8)		

In Table 2, the mean comparisons of variables including age, duration of diabetes, FBS, BMI, HbA1c, cholesterol, triglycerides, HDL, LDL, creatinine, systolic blood pressure, and diastolic blood pressure are presented between the neuropathy and non-neuropathy groups. The mean FBS and duration of diabetes were not homogeneous between the two groups. Patients with higher FBS and longer duration of diabetes had a higher likelihood of neuropathy.

**Table 2:** Comparison of mean age, clinical, and laboratory variables of diabetic patients by neuropathy status T: statistic of independent t test; FBS: fasting blood sugar; BMI: body mass index; LDL: low-density lipoprotein; HDL: high-density lipoprotein

Variables	Neuropathy+	Neuropathy-	T	P-value
	Mean (SD)	Mean (SD)		
Age	63.8 (9.45)	64.1 (11.70)	0.231	0.818
Duration of Diabetes	26.2 (2.41)	48.9 (2.01)	7.122	< 0.001
FBS	172.2 (55.17)	164.6 (54.76)	-1.971	0.048
BMI	28.4 (4.48)	28.5 (4.20)	0.310	0.757
Hb1Ac	8.2 (1.63)	7.7 (1.92)	-2.040	0.420
Cholesterol	201.0 (46.35)	192.4 (46.95)	-1.586	0.114
Triglyceride	196.7 (124.23)	193.1 (108.83)	-0.272	0.786
HDL	49.4 (22.02)	48.4 (16.89)	-0.404	0.687
LDL	112.6 (42.90)	110.2 (40.18)	-0.500	0.617
Creatinine	0.8 (0.24)	0.8 (0.29)	0.987	0.324

**Figure 1:**
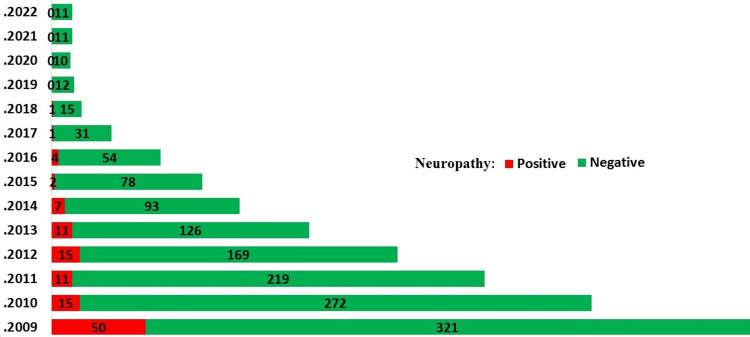
Trend of neuropathy incidence in type 2 diabetes patients over 11 years of follow-up

Figure 1 illustrates the frequency of neuropathy occurrence in the diabetic patients included in the study. The highest frequency of occurrence was observed in the early years following neuropathy diagnosis.

Simple logistic regression was performed with all covariates. Among them, gender, race, family history of diabetes, type of treatment, cholesterol, HDL, and duration of diabetes were significant at the 0.2 level and were included in the multiple logistic regression model fitting. The results of the final fitted multiple logistic regression model are presented in Table 3. Based on the results of the fitted logistic regression model, women have a 2.12 times higher chance of developing neuropathy compared to men. Individuals with a family history of diabetes have a 2.62 times higher chance of developing neuropathy compared to those without such a history. The odds ratio for patients using injectable medication is 4.32 compared to those using oral medication, and this ratio is 12.35 for those using both injectable and oral medications compared to the reference group. For each additional month of diabetes duration, the odds of developing neuropathy decrease by a factor of 0.98. In other words, an increase in the duration of diabetes is associated with a reduced chance of developing neuropathy.

**Table 3:** Results of multiple logistic regression for predicting neuropathy event

Variables	OR	P-value	95% CI	
			Lower	Upper
Gender				
Female	2.12	0.015	1.12	3.88
Male	1	-	-	-
Family History				
Yes	2.62	< 0.001	1.16	3.88
No	1	-	-	-
Treatment				
Oral	1	-	-	-
Injection	4.32	0.005	1.55	12.03
Both	12.35	< 0.001	4.44	34.36
Duration of Disease	0.96	< 0.001	0.95	0.98

**Figure 2:**
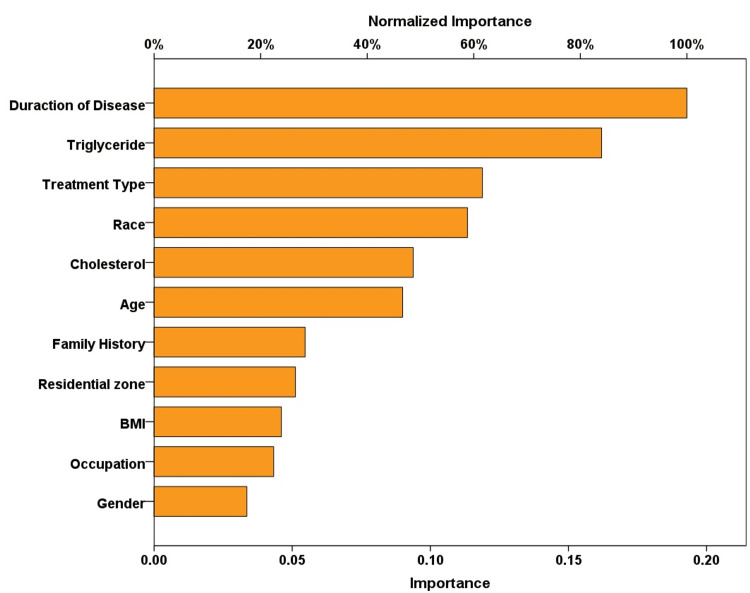
Importance of significant variables in the neural network for predicting neuropathy occurrence in type 2 diabetes patients

Figure 2 illustrates the results of the best-fitted neural network, displaying the highest area under the curve (AUC) value (0.903) and the lowest error rate. The significant variables, ranked by importance, included duration of diabetes, triglyceride levels, type of diabetes treatment, race, cholesterol levels, age, family history of diabetes, residential zone, BMI, occupation, and gender.

Comparison of the performance of the ANN and logistic regression

**Figure 3:**
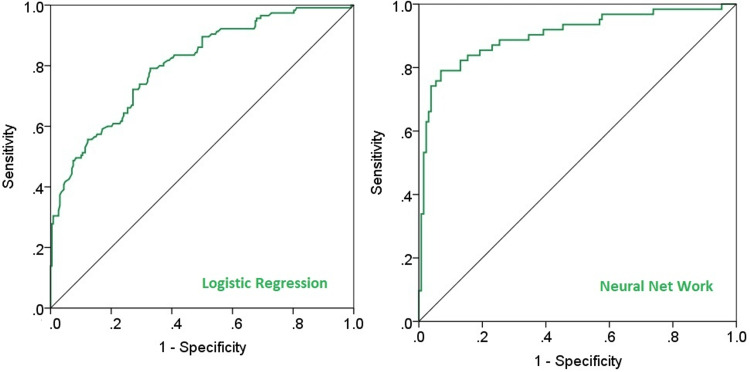
ROC curves of the artificial neural network and logistic regression models fitted for predicting neuropathy occurrence in type 2 diabetes patients ROC: receiver operating characteristic

The area under the receiver operating characteristic (ROC) curve for the two methods was calculated to be 0.903 and 0.804, respectively, indicating the superior performance of the neural network in identifying factors influencing neuropathy occurrence. Figure 3 shows the ROC graph for the two methods.

## Discussion

In the current study, the prevalence of neuropathy among patients with T2D in Fereydunshahr was estimated to be 31.2%, consistent with findings from global research. However, variability in neuropathy prevalence exists across studies, with reported rates ranging from 14.1% to 69%. Notable differences were observed in studies conducted by Muller (20-40%) [10], Yun et al. in Korea (14.1-54.5%) [11], and in the DiabCare-Asia study (34%) [12].

The higher risk of neuropathy in female patients with T2DM compared to males can be attributed to various factors. Firstly, hormonal differences between males and females may play a role. Estrogen, for instance, has been shown to influence nerve function and may contribute to the development or progression of neuropathy [13]. Age is a crucial variable in understanding the risk and progression of neuropathy in patients with T2DM. Aging is associated with physiological changes in the nervous system, including decreased nerve conduction velocity, axonal degeneration, and impaired nerve regeneration [13]. These age-related changes make older individuals more susceptible to neuropathic complications, including diabetic neuropathy. Moreover, older age is often accompanied by comorbidities such as hypertension, dyslipidemia, and obesity, which further exacerbate the risk of neuropathy in diabetic patients [14]. Additionally, the duration of diabetes, which tends to increase with age, is a significant determinant of neuropathy risk. Prolonged exposure to hyperglycemia leads to cumulative nerve damage, increasing the likelihood of neuropathic symptoms [15]. Furthermore, older patients may have decreased mobility and physical activity levels, which can exacerbate neuropathy symptoms and contribute to functional impairment [16]. Therefore, age serves as a critical predictor of neuropathy risk in patients with T2DM, highlighting the importance of age-specific interventions and management strategies to mitigate neuropathic complications.

Treatment type is a crucial factor influencing the occurrence of neuropathy in patients with T2DM. The mode of treatment can vary from oral medications to injectables, or a combination of both, depending on the severity of the diabetes condition. Each treatment modality may exert different effects on the development and progression of neuropathy. Research suggests that the method of treatment, particularly the use of injectable medications, may contribute to an increased risk of neuropathy in diabetic individuals. Additionally, factors such as medication adherence, dosage, and duration of treatment may also play a role in determining the likelihood of neuropathy development. Further investigation into the specific impact of different treatment types on neuropathy incidence is warranted to better understand their clinical implications and inform treatment strategies for diabetic patients [17].

Elevated levels of triglycerides and cholesterol have been found to be associated with the occurrence of neuropathy in our study. Research indicates that an increase in triglyceride and cholesterol levels can exacerbate neurological complications in individuals with T2DM [18]. Epidemiological studies have shown a correlation between elevated triglyceride and cholesterol levels and an increased risk of neuropathy in diabetic patients [19]. Furthermore, these factors can accelerate the progression of neuropathy and worsen neurological symptoms. Therefore, proper control of triglyceride and cholesterol levels may serve as an effective strategy for reducing the risk and expediting the treatment of neuropathy in individuals with T2DM.

In the present study, we found a significant association between a family history of diabetes and an increased risk of neuropathy among individuals with T2DM. This suggests that genetic factors contributing to diabetes susceptibility may also influence the development of neuropathic complications. The familial predisposition to diabetes could potentially indicate shared genetic or environmental risk factors that predispose individuals to both diabetes and neuropathy [20]. Understanding the role of family history in neuropathy risk can aid in identifying high-risk individuals and implementing targeted preventive strategies.

Overall, we utilized ANN and logistic regression in our study and observed that ANNs were more effective in identifying the factors influencing the occurrence of neuropathy in patients with T2DM. This superiority of ANNs has also been observed in other studies, where they have demonstrated higher accuracy and predictive power in medical diagnosis and prognosis compared to traditional statistical methods [21,22].

Limitations

The primary limitation encountered in this study was related to missing data. The extended duration of the follow-up period posed challenges in maintaining consistent access to study samples due to factors such as migration. Consequently, measures such as telephone outreach, text messaging, and home visits were undertaken to mitigate the rate of data loss. Another significant limitation is the geographic specificity of the study, conducted in Fereydunshahr, Iran. This specificity may impact the generalizability of our findings to other populations and regions. Although we employed advanced ANN methods and collected comprehensive data over a 12-year follow-up period to enhance the accuracy and predictive capability of our results, the unique demographic, cultural, and environmental characteristics of this area may have uniquely influenced the outcomes. Therefore, we recommend that similar studies be conducted in diverse geographic locations with varying population characteristics to improve the validity and generalizability of the findings.

## Conclusions

The study outcomes shed light on the crucial factors influencing neuropathy development in patients with T2DM. We observed notable disparities in demographic and clinical variables between individuals with and without neuropathy. Gender, ethnicity, family history of diabetes, treatment modality, cholesterol levels, HDL, and diabetes duration emerged as significant predictors. Additionally, the superior predictive performance of ANN over logistic regression underscores the potential of advanced machine learning techniques in enhancing neuropathy prediction accuracy. The neural network demonstrated enhanced performance compared to logistic regression, highlighting its effectiveness in predicting neuropathy occurrence. These findings underscore the importance of early identification and targeted interventions for at-risk diabetic patients to mitigate neuropathy onset and progression.
